# An S-adenosyl Methionine Synthetase (SAMS) Gene from *Andropogon virginicus* L. Confers Aluminum Stress Tolerance and Facilitates Epigenetic Gene Regulation in *Arabidopsis thaliana*

**DOI:** 10.3389/fpls.2016.01627

**Published:** 2016-11-08

**Authors:** Bunichi Ezaki, Aiko Higashi, Norie Nanba, Takumi Nishiuchi

**Affiliations:** ^1^Institute of Plant Science and Resources, Okayama UniversityKurashiki, Japan; ^2^Institute for Gene Research, Kanazawa UniversityKanazawa, Japan

**Keywords:** aluminum (Al) stress, *AvSAMS1* gene, DNA methylation, epigenetic gene-regulation, histone H3 methylation, microarray analysis, multiple abiotic stress tolerance

## Abstract

Candidate clones which conferred Al tolerance to yeast transformants (TFs) were obtained from a cDNA library derived from a highly Al-tolerant poaceae, *Andropogon virginicus* L. One such clone, AL3A-4, encoded an S-adenosyl methionine synthetase (SAMS) gene. A full-length cDNA was obtained by 5′-RACE, designated *AvSAMS1*, and introduced into *Arabidopsis thaliana* to investigate its biological functions under Al stress. Two TF plant lines both showed higher tolerance than the Col-0 ecotype (non-TF) not only for Al stress, but also for Cu, Pb, Zn and diamide stresses, suggesting the *AvSAMS1* was a multiple tolerance gene. More than 40 of *A. thaliana* Al response-genes (Al induced genes and Al repressed genes) were selected from microarray results and then used for investigations of DNA or histone methylation status under Al stress in Col-0 and the *AvSAMS1* TF line. The results indicated that Al stress caused alterations of methylation status in both DNA and histone H3 (H3K4me3 and H3K9me3) and that these alterations were different between the *AvSAMS1* TF and Col-0, suggesting the differences were *AvSAMS1*-gene dependent. These results suggested the existence of *AvSAMS1*-related epigenetic gene-regulation under Al stress.

## Introduction

Aluminum (Al) in acid soil areas is solubilized into soil solution below pH 5.0 as a toxic form, Al^3+^, and its toxicity results in a loss of crop yields as a limiting factor of plant growth in agriculture. The root apex is a primary target of Al toxicity and an inhibition of root growth is the major symptom of Al toxicity in plants. Secretion of organic acid anions, such as malate, oxalate, citrate and succinate, from root tips into soil has been considered as one of the most effective Al tolerance strategies (Delhaize et al., 1993). Sasaki et al. (2004) isolated the *ALMT1* gene encoding a malate transporter from *Triticum aestivum* and showed that this gene confers Al tolerance in transgenic tobacco cells. Plasma-membrane-localized Al transporter proteins, Nrat1 and PALT1, were isolated from *Oryza sativa* and *Hydrangea macrophylla*, respectively (Xia et al., 2010; Negishi et al., 2012). Recently, Ezaki et al. (2015) reported an Al stress induced half type ABCG transporter protein derived from a poaceae wild plant, *Andropogon virginicus* L. These proteins are suggested to decrease toxic cytoplasmic Al by their transport systems. It has been also demonstrated that induction of anti-peroxidation enzymes can ameliorate the oxidative damage caused by Al stress and lead to Al tolerance phenotypes in various plants (Richards et al., 1998; Ezaki et al., 2001; Milla et al., 2002; Boscolo et al., 2003; Watt, 2003). Phenolic compounds, such as flavonoids, alkaloids, terpenoids and glycosides, form strong complexes with toxic Al ions and are implicated in internal Al detoxification especially in Al-accumulating species (Kidd et al., 2001; Ofei-Manu et al., 2001; Ito et al., 2009).

Many wild plants show extremely high tolerance against abiotic stresses, such as salinity, metal toxicities, drought, temperature stresses and oxidative stresses (Ellis et al., 2000; Ivandic et al., 2000; Akashi et al., 2001; Bartels, 2001; Shen et al., 2001; Mittova et al., 2004). We reported that *A. virginicus* L. shows a high Al tolerance by a combination of five independent tolerance mechanisms including a low Al accumulation in its root-tip region by the secretion of organic acids and inductions of NO, poly-phenols and anti-peroxidation enzymes such as superoxide dismutase (EC1.15.1.1) and catalase (EC 1.11.1.6) (Ezaki et al., 2013). It was also very interesting that most of the Al tolerance mechanisms of this plant were Al inducible, suggesting a systemic Al-induced mechanism in the expression of these genes. However, the precise gene regulation system involved in Al tolerance has not yet been characterized.

Recently epigenetic gene regulation has been well studied and this wide ranging system is related to adaptation for various environmental stresses in plants via methylations of DNA, RNA and histones as well as other modifications of histones (Hauser et al., 2011; Kou et al., 2011; Wang et al., 2011; Gayacharan and Joel, 2013; Garg et al., 2015). Therefore, methylation is thought to be a very important modification in epigenetic regulation. S-adenosyl methionine (SAM), synthesized from methionine and ATP by SAM synthetase (SAMS; EC2.5.1.6), is involved as the main methyl group donor in many organisms including plants. Highly reactive methyl residue of SAM is very useful for methylation of DNA, RNA, protein, lignin, flavonoid and so on, and it also plays important roles in regulating plant development, abiotic or biotic stress, and metabolic accumulation (Xu et al., 2006; Nagel et al., 2008; Köllner et al., 2010). It is also well known that SAM is used as a precursor in the biosynthesis of polyamines and ethylene in plants (Pandey et al., 2000; Roje, 2006). *OsSAMS1*-RNAi transgenic plants with repressed transcripts of all three *OsSAMS* genes (*OsSAMS1, OsSAMS2*, and *OsSAMS3*) exhibited a severe late-flowering phenotype (Li et al., 2011). They suggested that the alterations in H3K4 tri-methylation and DNA methylation at specific genes suppressed their expression and subsequently led to late flowering and concluded that SAM, as a major methyl donor, plays a critical role in the epigenetic control of flowering.

In this study, we isolated an Al tolerance gene, *AvSAMS1* from *A. virginicus* L. The *AvSAMS1* dependent epigenetic gene-regulation was facilitated under Al stress in an *A. thaliana* transformant (TF) expressing this gene. This is the first report demonstrating a biological relationship between a *SAMS* gene, Al tolerance and a deduced epigenetic gene regulation in the TF.

## Materials and methods

### Growth conditions of plants and yeast

*A. virginicus* L., *A. thaliana* Col-0 ecotype and its derivatives were used in this study. Seeds of *A. virginicus* L. were submerged in distilled water for 7 to 10 days at 4°C and then grown in hydroponic conditions with a nylon mesh net and a floating supporter to keep the seeds at an adequate water level in 0.5 mM CaCl_2_ solution (adjusted to pH 5.7). Approximately 2- to 3-week-old seedlings were used for each experiment. For *A. thaliana*, seeds were submerged in distilled water for 4 days at 4°C, grown in soil for approximately 3 week and then used for all experiments except for stress sensitivity tests. For stress sensitivity tests, seeds of *A. thaliana* were sterilized, submerged in sterilized water for 4 days at 4°C and then grown in hydroponic conditions with a fine nylon mesh net and a floating support in 1/6 MS medium (adjusted to pH 5.7; Ezaki et al., 2000) for 12 to 15 days. All plants were grown under fluorescent illumination (approximately 50 μEm^−2^s^−1^, 16 h of light and 8 h of darkness) at 25°C.

*Saccharomyces cerevisiae* strain, INVSc1 (Invitrogen, USA), was used for a direct screening of the Al tolerant genes derived from *A. virginicu*s L. YNB (yeast nitrogen base) agar plate (1.5% agar in YNB liquid medium) adjusted to pH 5.7 were used for yeast growth and all stress-sensitivity-tests except for Al stress. For Al stress-sensitivity-test, a low phosphate and magnesium (LPM) agar plate (1.5% agar in LPM liquid medium) adjusted to pH 4.2 was used (MacDiarmid and Gardner, 1998). Yeast TF cells were grown at 30°C for 3 to 4 days to estimate their sensitivities to various stresses.

### Abiotic stresses for plants and yeast

Seedlings of *A. virginicus* L. (2- to 3-week-old) were transferred to fresh 0.5 mM CaCl_2_ solution (adjusted to pH 4.2) including 0 or 300 μM AlCl_3_ for 8 or 24 h to investigate their gene-expressions. Young seedlings were also exposed to 35 μM CdCl_2_, 50 μM CuSO_4_, 300 μM PbCl_3_, 200 μM ZnCl_2_, 1.5 mM diamide (DM; 1,1′-Azobis (*N*,*N*-dimethyl formamide), Sigma-Aldrich, USA) or 1.5 mM H_2_O_2_ in 0.5 mM CaCl_2_ solution at pH 5.7 for 8 h to determine gene-expression in each condition. Seedlings of *A. thaliana* grown in 1/6 MS medium (10-days to 2-weeks-old) were also exposed to 300 μM AlCl_3_ in a fresh 1/6 MS medium (adjusted to pH 4.2) for 2 days or to 35 μM CdCl_2_, 50 μM CuSO_4_, 300 μM PbCl_3_, 200 μM ZnCl_2_, 1.5 mM DM or 1.5 mM H_2_O_2_ in a fresh 1/6 MS medium (adjusted to pH 5.7) for 2 days. Root length was randomly measured for more than 20 plants in each condition to calculate the relative root growth which was shown previously (Ezaki et al., 2000).

### Direct screening of Al tolerant genes from *A. virginicus* L.

Approximately 2- to 3-week-old seedlings of *A. virginicus* L. grown in hydroponic condition (0.5 mM CaCl_2_, pH 5.7) were transferred to 0.5 mM CaCl_2_ medium (pH 4.2) including 300 μM Al for another 24 h. Total RNA was extracted from the Al-treated plants using RNeasy Plant Mini Kit (Qiagen, USA) and then purified by Fast Track MAG mRNA isolation Kits (Invitrogen) to obtain poly(A)^+^-RNA molecules. Double stranded cDNA molecules were moreover prepared by a cDNA Synthesis Kit (M-MLV version) (TAKARA, Japan) using the extracted poly(A)^+^-RNA and then both ends of these cDNAs were blunted. A Yep-type vector, pYES3 (Smith et al., 1995), were digested with *Eco*RI and then blunted by T4 DNA polymerase. These blunted cDNAs and the vector DNA were mixed, ligated and introduced into *Escherichia coli* to amplify them once. The pool of extracted plasmids from the *E. coli* transformants were used as a cDNA library (DNA donor) for yeast transformation and direct selection on LPM agar plates containing 300 μM Al. CapFishing Full-Length cDNA Premix Kit (Seegene, USA) was used for 5′-RACE to obtain a full length clone of the Al tolerant gene.

### Construction of *A. thaliana* transgenic lines

The full-length cDNA fragment of *AvSAMS1* was inserted between the cauliflower mosaic virus (CaMV) 35S promoter and the octopine synthase gene (*OCS*) terminator in pART7 and then cloned into the *Not*I site of pART27 (Gleave, 1992). The construct was introduced into *Agrobacterium tumefaciens* LBA4404 to get kanamycin-resistant (Km-r) TFs. Transformation of *A. thaliana* (Col-0 ecotype) by *A. tumefaciens* TFs was performed by a dipping method (Clough and Bent, 1998). Five times of dipping (2 or 3 plants per one dipping) were performed in the *A. thaliana* transgenic events. T1 transgenic lines were selected on the 1/6 MS plates (adjusted to pH5.7) containing 1% agar and 75 mg L^−1^ Km and more than 30 of Km-r TFs were isolated. The Km-r seedlings were transferred to soil and grown to maturation. Screening of seeds for Km-r progeny was carried out on the same medium to get single-gene inserted, homozygous T2 transgenic lines and five independent TF lines were finally obtained. These TF lines were tested their gene-expression level of the *AvSAMS1* by qRT-PCR. Two highest expressing TF lines, TF3 and TF6, were used for abiotic stress sensitivity tests. And TF6 was used for microarray analysis and DNA and histone methylation analyses.

### Microarray analysis

After the Al treatment (0 or 300 μM Al for 2 days) of the *AvSAMS1*-expressing *A. thaliana* TF and its parental non-TF, Col-0 line, total RNA were extracted from these four plant samples by RNeasy Plant Mini Kit (Qiagen) and their quality was confirmed by the RIN (RNA Integrity Number) method (Agilent Technologies, USA). Two independent sets of RNA from the different biological pools of plant samples (Al-treated Col-0, untreated Col-0, Al-treated *AvSAMS*1 TF6 and untreated *AvSAMS1* TF6) were subjected to the total four 2-color microarray analyses. Total RNA samples were labeled independently with Cy3 (untreated) or Cy5 (Al-treated), respectively using Quick Amp Labeling Kit, Two-color (Agilent Technologies). A 4 X 44 K format microarray for *A. thaliana* Ver4.0 (G2519F#21169, Agilent Technologies) was used for hybridization in this experiment. Equal amounts of the two labeled cRNAs were mixed and competitively hybridized on the same slide at 65°C for 17 h. Agilent Feature Extraction and Agilent GeneSpring GX ver.12.5 (Agilent Technologies) were used for analyses. Total four microarray analyses were performed for a screening of Al response genes (Al induced genes and Al repressed genes) from Col-0 and the *AvSAMS1* TF line as follows;

Array:252116911539_1_1, Cy3: untreated Col-0#1, Cy5: Al-treated Col-0#1
Array:252116912697_1_1, Cy3: untreated Col-0#2, Cy5: Al-treated Col-0#2
Array:252116911538_1_4, Cy3: untreated AvSAMS1TF6#1, Cy5: Al-treated AvSAMS1TF6#1 Array:252116912697_1_3, Cy3: untreated AvSAMS1TF6#2, Cy5: Al-treated AvSAMS1TF6#2.

Means value of the two relative holds of each gene-expression under +Al condition to –Al condition in the two independent microarray analyses were compared between Col-0 and the *AvSAMS1* TF line. Then, we showed the results of *P*-value (<0.05) of LogRatio(+Al/–Al). These selected genes were confirmed their expression patterns by qRT-PCR, if necessary. They were moreover used for investigation of methylation status of DNA or histone under Al stress in this study. The microarray data was deposited in GEO database (accession number: GSE85593).

### DNA sequencing after bisulphite treatment

To investigate alterations of DNA methylation by Al treatment in Col-0 and the *AvSAMS1* TF, the genomic DNAs extracted from either Al-treated plants (300 μM Al treatment for 2 days) or untreated plants (0 days) were subjected to bisulphite treatment using a rapid DNA bisulphite modification kit, MethyEasy Xceed (Human Genetic Signatures Pty Ltd., USA) and then applied to DNA sequencing. The Methyl Primer Express® Software v1.0 (supplied from Applied Biosystems, USA) was used to design primers for methylation-focused PCR experiments.

### Chromatin immuno-precipitation (ChIP) analysis

To investigate alterations of histone3 (H3) methylation status under Al stress, three independent chromatin preparations were performed for the four plant samples (Al-treated Col-0, untreated Col-0, Al-treated *AvSAMS*1 TF and untreated *AvSAMS1* TF). Genomic DNA fragments specifically associating with either tri-methylated H3K4 (H3K4me3) or tri-methylated H3K9 (H3K9me3) were isolated from the immune-precipitated chromatin according the manual described previously with a minor modification (Villar and Köhler, 2010). Rabbit anti-H3K4me3 antibody and anti-H3K9me3 antibody were used for immuno-precipitation (Active Motif, USA) in this ChIP assay. To calculate the relative enrichment of the target DNA in the immuno-precipitated chromatin fractions including either H3K4me3 or H3K9me3, contents of the recovered target DNA fragments were determined by qRT-PCR, using LightCycler 1.5 (Roche, Germany) and SYBR Premix Ex TaqII (TAKARA). Three sets of primers were individually designed to amplify the target fragments #1, #2, and #3 (see **Figures 5B,D**) and used for the qRT-PCR. DNA contents of the three target fragments in eight nucleosome fractions (four nucleosome fractions extracted from Al-treated Col-0 line or non-treated Col-0 line and then precipitated with anti-H3K4me3 antibody or anti-H3K9me3 antibody, and another four nucleosome fractions extracted from Al-treated or non-treated *AvSAMS1* TF line and then precipitated with either of the two antibodies shown above) were normalized to an internal control, *AtAct1* gene of *A. thaliana* (At2g37620). At least three replicates of qRT-PCR experiments were performed for each DNA fragment.

### Statistical analysis

Experiments included two or three independent replicates. In case of the three independent replicates, means of the obtained values were calculated along with standard deviations (SD). *F*-tests were performed to identify differences in variance between the two tested groups. Then either the Student's *t*-test (when variances were similar) or Welch's *t*-test (when variances were different) was applied to calculate the significance of differences between the means.

## Results

### Direct screening of Al tolerant genes from *A. virginicus* L.

**Figure 1:**
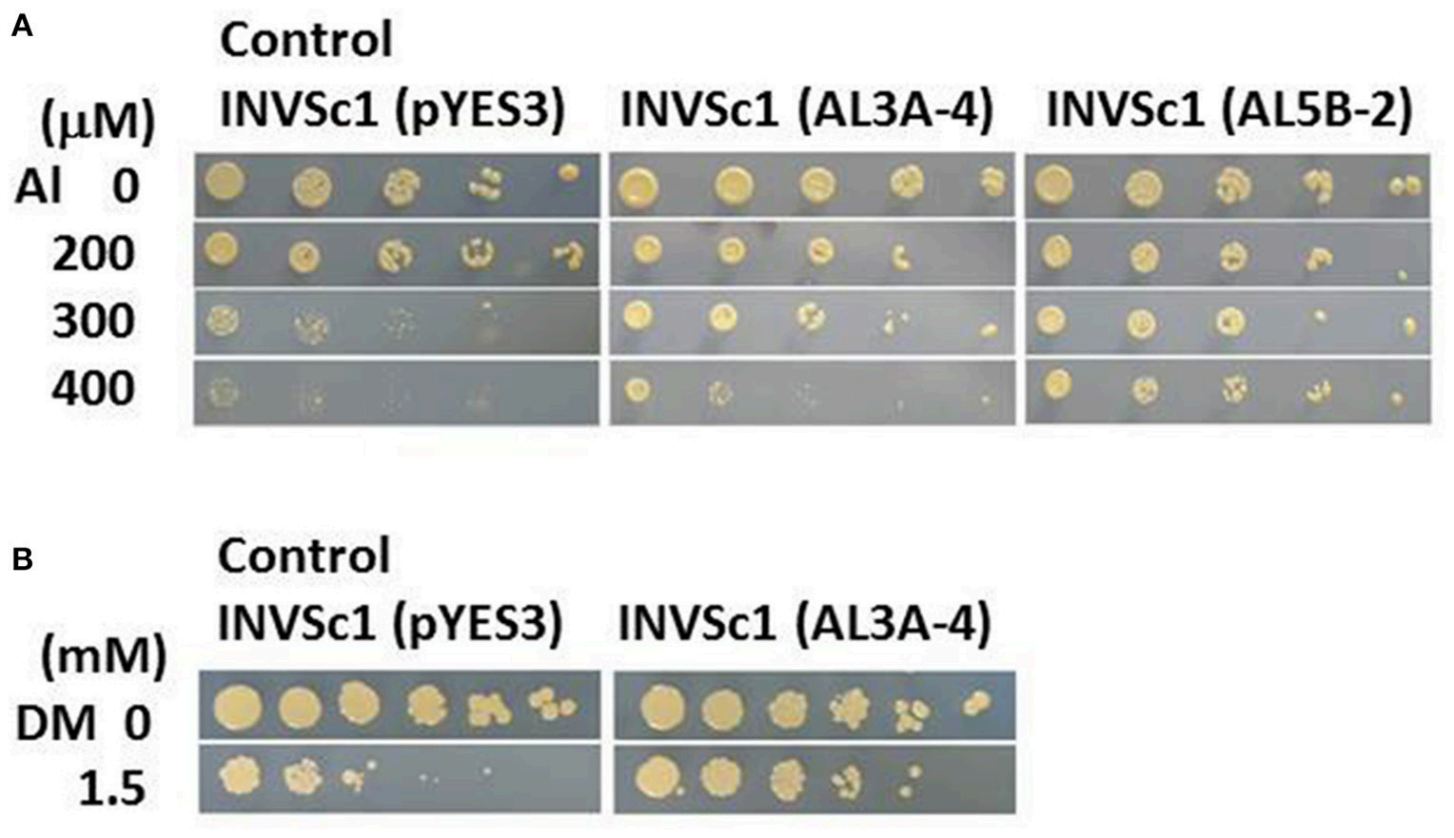
**Sensitivity test of the yeast TF carrying AL3A-4 or AL5B-2 clones on agar plates**. **(A)** Al test on LPM agar plate (adjusted at pH4.2), **(B)** DM test on YNB agar plate (adjusted at pH5.7). Log phase cells of the TFs carrying either pYES3 (vector), AL3A-4 or AL5B-2 were serially diluted with sterilized water(1/5~1/3125 fold for Al test and 1~1/3125 fold for DM test) and then spotted on the agar plates. Plates were incubated at 30°C for 3 to 4 days and the sensitivity was estimated by a colony forming ability on the agar plates.

Wild plant *A. virginicus* L. shows a high Al tolerant phenotype by a combination of five Al tolerant mechanisms at least in our previous study (Ezaki et al., 2013). To characterize its high Al tolerance mechanism from molecular genetic points of view, Al tolerant genes derived from this plant were tried to isolate from a full length cDNA library, using *S. serevisiae* (yeast) INVSc1 as a screening strain. Pool of cDNA clones carrying the *A. virginicus* L. cDNA were introduced into INVSc1 and then the *ura*^+^ and Al tolerant TFs were directly screened on the LPM agar plates including 300 μM Al. Two candidate clones (AL3A-4 and AL5B-2) were obtained in the first screening and their tolerance to Al toxicity was confirmed. Compared with the yeast INVSc1 TF carrying a vector, pYES3, these two TFs showed a higher growth on the 300 μM Al agar plates (Figure 1A). Moreover the AL5B-2 could form colonies on 400 μM Al agar plate. Plasmid DNAs were extracted from these two tolerant TFs and the inserted cDNA fragments were individually sequenced. Result of a primitive homology search of these two clones to DDBJ (DNA Data Bank of Japan) indicated that the AL3A-4 was an S-adenosyl methionine (SAM) synthetase (SAMS) gene (approximately 90% identity in DNA sequence with *O. sativa* AK103157 and with *Zea mays* BT088116)(data not shown). The other clone, AL5B-2, had a homology to a *Z. mays* function-unknown clone, DY620452 with approximately 90% homology (data not shown). We therefore focused on AL3A-4 in further analyses in this study. The yeast TF carrying the AL3A-4 was furthermore exposed to various heavy metal stresses, such as Cu, Cd, Pb and Zn, and to two oxidative stresses, DM and H_2_O_2_. It did not show a clear tolerance to all of these stresses except for DM stress in yeast TF (Figure 1B).

### Property of *AvSAMS1* gene

Since the primitive comparison of the DNA sequence among AL3A-4 clone and the other *SAMS* genes indicated that this clone lacked the 5'-end, a full length of cDNA was tried to isolate by 5'-RACE. The longest clone completely carried its N-terminal region and 29-nt-upstream region of ATG start codon. Highest homology (95% identity) in DNA sequence was seen between the *SAMS* gene of *Saccharum hybrid* cultivar (KJ577596) and other high homologies were also seen among those of *O. sativa* and *Z. mays* described above (90 and 94% identity, respectively) (Supplement Figure 1). The *SAMS* gene derived from *A. virginicus* L. was therefore designated as *AvSAMS1* gene (accession number AB907169) and used for further analyses in this study. Open reading frame of the *AvSAMS1* gene was 1188 nt encoding 396 amino acids and the deduced molecular weight was 98.81 kDa. A high homology in amino acid sequence was also observed in a wide range of organisms (Supplement Figure 2).

**Figure 2:**
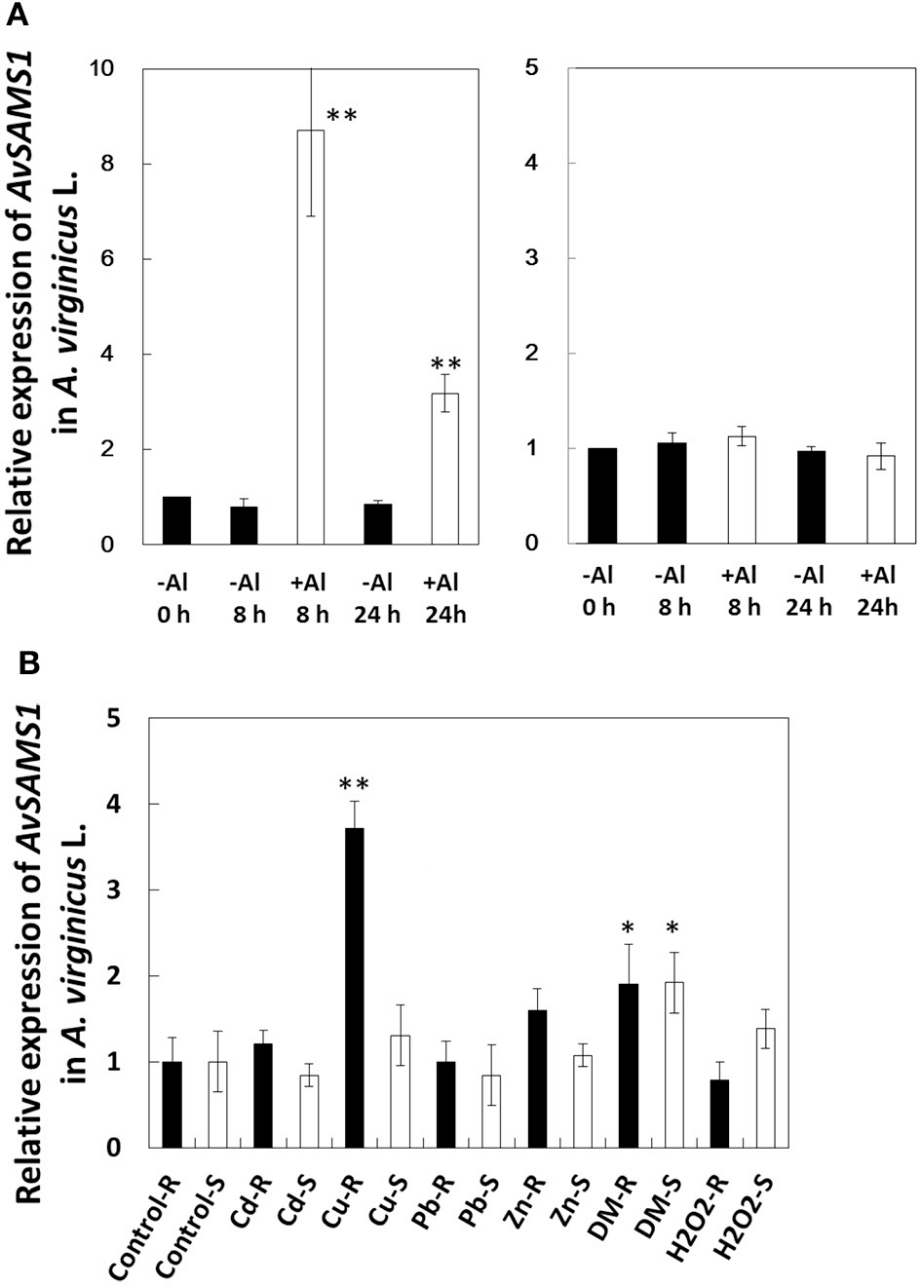
**Gene-expression profile of *AvSAMS1* in *A. virginicus* L. (A)** Al. Gene expression in root (left) and shoot (right) are represented as expression of the *AvSAMS1* relative to each control condition (-Al, 0 h). **(B)** Cd, Cu, Pb, Zn, DM, and H_2_O_2_. Gene-expression under each tested condition in roots (R) and shoots (S) are also shown as relative expression to the control conditions (Control-R and Control-S, respectively). Error bars indicate standard deviations (SD). Asterisks indicate significant differences to each control (^*^p < 0.05, ^**^p < 0.01).

The transcriptional response of the *AvSAMS1* gene to Al stress in *A. virginicus* L. was precisely confirmed by qRT-PCR (Figure 2A). Compared with the basal expression level of this gene under non-treated conditions (0 h), approximately 8.8-fold higher expression was occurred in roots after an exposure to 300 μM Al stress for 8 h and this elevated expression was maintained at 24 h (approximately 3.4-fold), whereas the expression was very low in shoots during the Al treatment (Figure 2A). Young seedlings of *A. virginicus* L. were also exposed to several abiotic stresses, such as Cd, Cu, Pb, Zn, H_2_O_2_ and DM for 8 h and the transcriptional responses to these stressors in root and shoot were determined (Figure 2B). The results indicated that the *AvSAMS1* was induced in roots by Cu and slightly by DM stress (approximately 3.7 and 1.9-fold, respectively). DM dependent induction was also detected in shoots (approximately 1.9-fold).

### *AvSAMS1* is a multiple tolerant gene for metal stress and oxidative stress

**Figure 3:**
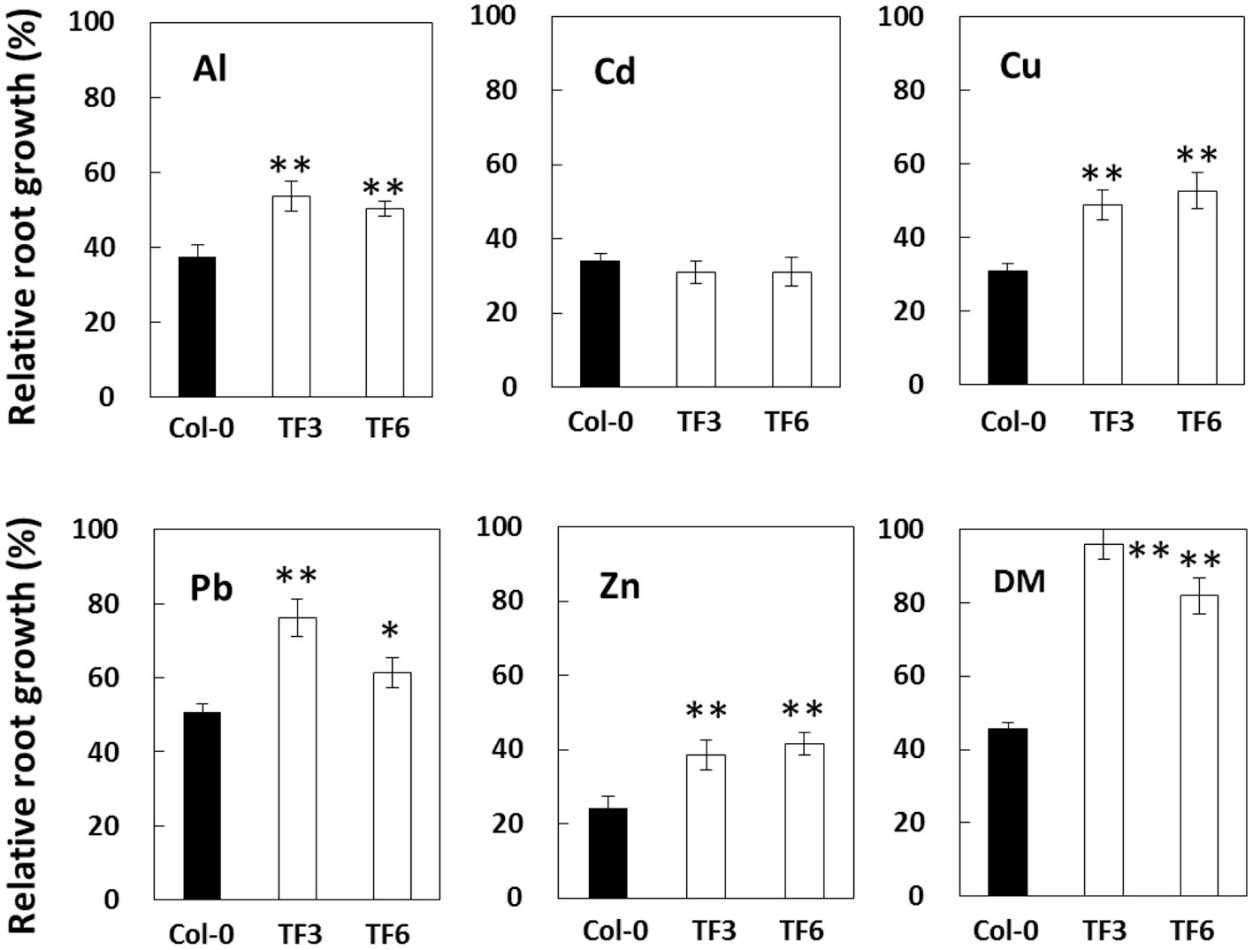
**Results of stress sensitivity tests in *A. thaliana* TF**. Two of the constructed *A. thaliana* TF expressing the *AvSAMS1* gene (TF3 and TF6) and their parental non-TF line, Col-0, were treated with various stressors and then measured for root length. Results were shown as relative root growth (%) as described previously (Ezaki et al., 2000). Error bars indicate SD. Asterisks indicate significant differences to each control shown as black bar (^*^p < 0.05, ^**^p < 0.01).

To characterize the biological functions of this gene under Al stress and other stresses in plant, transgenic *A. thaliana* carrying the *AvSAMS1* were constructed and two of single-copy-inserted homozygote TFs were obtained (TF3 and TF6). These two plant lines grew and flowered normally, with no visible phenotypic difference from wild-type Col-0. Sensitivity tests for various stressors (Al, Cd, Cu, Pb, Zn, and DM) were performed and both of the TF lines showed higher relative root growth than a control Col-0 ecotype under Al, Cu, Pb, Zn, and DM treatments, but not under Cd stress (Figure 3). These results suggested that the *AvSAMS1* gene could confer multiple stress tolerance in *A. thaliana*.

### Microarray analysis

It is well known that SAM is widely used as a methyl residue donor in many metabolisms, including the methylation processes of both DNA and histone. It is also well known that a genome-wide alteration in gene-expression (repression and induction), so-called epigenetic gene-regulation, can be led by these methylation processes (see review; Henderson and Jacobsen, 2007). It was therefore hypothesized that a highly expressed AvSAMS1 enzyme might accumulate higher amounts of SAM and preferentially promote a higher epigenetic gene regulation in the *AvSAMS1* TF line, compared with Col-0 ecotype. To prove this hypothesis, methylation status of genome DNA and histone protein should be determined and compared between the two lines [“Al-treated Col-0 vs. untreated Col-0” and “Al-treated *AvSAMS*1 TF vs. untreated *AvSAMS1* TF”], using Al response genes as target genes. To select such target genes, microarray analysis was performed and the gene-expression patterns with and without Al were compared in the two lines [“Al-treated Col-0 vs. untreated Col-0” and “Al-treated *AvSAMS*1 TF vs. untreated *AvSAMS1* TF”] (Supplement Figure 3). From genes induced (more than approximately 2-fold) or repressed (less than approximately 0.5-fold) by Al stress, two groups were defined that were affected in both or either plant lines and that were also more highly induced or more severely repressed in the *AvSAMS1* TF line than in Col-0 line. These two groups were classified and designated as “Gene group I (induced type)” or “Gene group R (repressed type)”, respectively and used for the investigation of methylation status in genome DNA and histone under Al stress.

### Methylation in DNA under Al stress

Six genes and eight genes were selected from the Gene group I and Gene group R, respectively and then applied to DNA sequencing based on a methylation-focused PCR. Results are summarized in Table 1. As a primary result of epigenetic gene-regulation, DNA methylation generally occurs in CG, CHG, or CHH sites in plant genomic DNA (H stands for A, C, or T). Approximately half of the tested regions showed non-methylation in all such C sites and no change in methylation status by Al treatment in either Col-0 or *AvSAMS1* TF plant. One representative result of the complete non-methylated status observed in At1g17180 (Glutathione transferase belonging to the tau class; Gene group I) is shown in Supplement Figure 4.

**Table 1:** **List of genes used for DNA methylation status analysis**.

**Gene**	**Biological function**	**Al response in Col-0^a^**	**Al response in TF^b^**	**Response type (Gene group)^c^**	**Methylation status^d^**	**Sequencing region (nt)**
Atlg07400	Class I Heat shock protein (HSP20)	3.52^*^	5.28^*^	I	non-ME	–187 ~+213
Atlgl7180	Glutathione transferase belonging to the tau class	6.08^*^	11.53^*^	I	non-ME	–180 ~+130
Atlgl7710	Pyridoxal phosphate phosphatase-related protein	2.66^*^	4.95^*^	I	ME	–278 ~+164
At2g34430	Photosystem 11 type I chlorophyll a/b-binding protein	0.46^*^	1.26^*^	I	ME	–160 ~+40
At3g06435	Uncharacterized protein	1.35	2.05^*^	I	non-ME	–388 ~+77
At3g56060	Glucose-methanol-choline (GMC) oxidoreductase family protein	0.71	0.22^*^	R	ME	–263 ~+209
At4gl2230	alpha/beta-Hydrolases superfamily protein	0.50	0.18^*^	R	ME	+123 ~+407
At4gl8940	RNA ligase/cyclic nucleotide phosphodiesterase family protein	1.08	0.07^*^	R	ME	+57 ~+452
At4g24310	Uncharacterized protein	0.39^*^	0.16^*^	R	ME	–172 ~+82
At4g33070	Pyruvate decarboxylase	1.01^*^	5.84^*^	I	non-ME	+1 ~+120
At5g07390(site 1)	Respiratory burst oxidase homolog A (RBOHA)	0.56	0.23^*^	R	non-ME	–263 ~+137
At5g07390(site2)					ME	+233 ~+593
At5g26280	TRAF-like family protein	0.39	0.15^*^	R	non-ME	–276 ~+109
At5g43370(site 1)	Phosphate transporter Phtl;2	0.35^*^	0.14^*^	R	ME	–228 ~ –43
At5g43370(site2)					non-ME	+296 ~+456
At5g63600	A protein similar to flavonol synthase	0.51	0.29^*^	R	non-ME	+736 ~+866

a Values indicated means of the two relative holds of each gene-expression in Col-0 ecotype under +A1 condition to −Al condition in the two microarray analyses.

b Values indicated means of the two relative holds of each gene-expression in the AvSAMSl TF line under +A1 condition to −Al condition in the two microarray analyses.

c I, Gene group I; R, Gene group R.

d non-ME, no-methyktion status was Al independently kept in both lines; ME, methylation status was altered in either the tested lines under Al stress.

* P-value of LogRatio (+AI/−AI) was less than 0.05.

**Figure 4:**
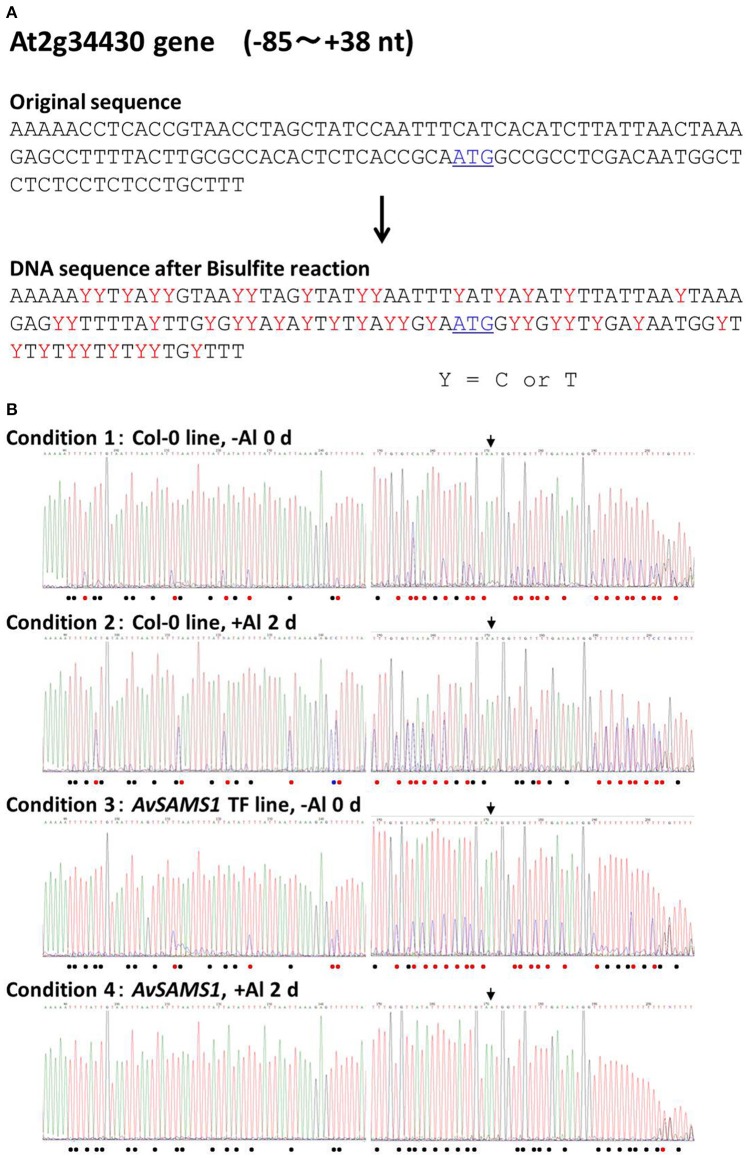
**Alteration of Al stress dependent DNA methylation status caused in the Al response gene, At2g34430. (A)** Comparison of the original DNA sequence and the deduced DNA sequence of At2g34430 after a bisulphite treatment. Y with red color in the latter sequence represents a mixture of C and T in the sequence, indicating both methylated C (retained as C after bisulphite treatment) and non-methylated C (converted to T by bisulphite treatment). The start codon, ATG, is shown by blue color and with underline. **(B)** Raw data of DNA sequence patterns of the four templates (Condition 1-4). Template DNAs (genomic DNA) were extracted from the untreated plants (0 days) (Condition 1 and 3) or from the Al treated plants (300 μM Al treatment for 2 days) (condition 2 and 4). Condition 1 and 2, Col-0 ecotype; Condition 3 and 4, the *AvSAMS1* TF line. Symbols shown in the bottom of each sequence pattern represented methylation status at C sites. •, Mixture of methylated C and non-methylated C; •, non-methylated C only; •, methylated C only. The limit for defining mixtures was taken as a ratio of >0.1 up to <0.9. Start codon, ATG, is shown by the arrow.

The remainder of the tested genes showed alterations of their methylation status under Al stress in either or both of the two lines. In case of At2g34430 (Photosystem II type I chlorophyll a/b-binding protein) belonging to Gene group I, there were 43 of C sites in the −85 to +38 nt region of the original DNA sequence (Figure 4A). The raw data of DNA sequencing are shown in Figure 4B. The effect of Al stress on Col-0 can be seen in the comparison of the top two electropherograms. The total number of single “T” peaks (shown by black dots in Figure 4B) indicating non-methylated C (which were completely converted to T by the bisulphite treatment) in Col-0 was increased from 15 to 20 by the Al treatment, indicating that there was an overall demethylation across the region. However, the situation was more complex, since some unmethylated sites also became partly methylated under Al stress, and one site (marked by the blue dot) switched from fully methylated to completely unmethylated. If we consider only the double peaks consisting of “T” and “C” in the same position in Col-0 line (shown by red dots in Figure 4B, representing a mixture of non-methylated C and methylated C), the ratios of the methylated to non-methylated C's under the +Al stress condition were clearly higher than the ratios at the same sites under −Al condition. This result suggested that increased methylation was occurring in these double-peak sites by Al stress. Taken together the results suggested that Al stress induced an intricate pattern of changes involving both demethylation and methylation, at C residues in the upstream region of At2g34430 in Col-0. Meanwhile, in the *AvSAMS1* TF line, 22 of the original C sites in the same region (shown by red dots) were altered to mixtures of methylated C and non-methylated C under −Al condition in the *AvSAMS1* TF line like those in Col-0 line (Figure 4B). However, under +Al condition, all of these double peaks except one were changed to T single peaks (shown by black dots), suggesting that most of the methylated C in these C sites were completely demethylated during Al stress in the TF line. These results show a dramatic difference in methylation status has been induced in the TF line. In case of At4g12230 (alpha/beta-Hydrolases superfamily protein) belonging to Gene group R, a clear difference of DNA methylation status between the two lines was also observed in the gene-body region far away from the start codon (195–247 nt from ATG site) (Supplement Figure 5). Eighteen of C sites in the original DNA sequence were mixture of methylated and non-methylated C (shown by red dots) under –Al conditions in the Col-0 line, most of which were changed to the methylated form (shown by blue dots) under +Al stress. In contrast, most of these C sites were non-methylated status under both –Al and +Al stress in the *AvSAMS1* TF line (shown by black bots). This result also indicated that the alterations occurred not only in the promoter region, but also in various positions of the coding region.

### Methylation in histone H3 under Al stress

**Figure 5:**
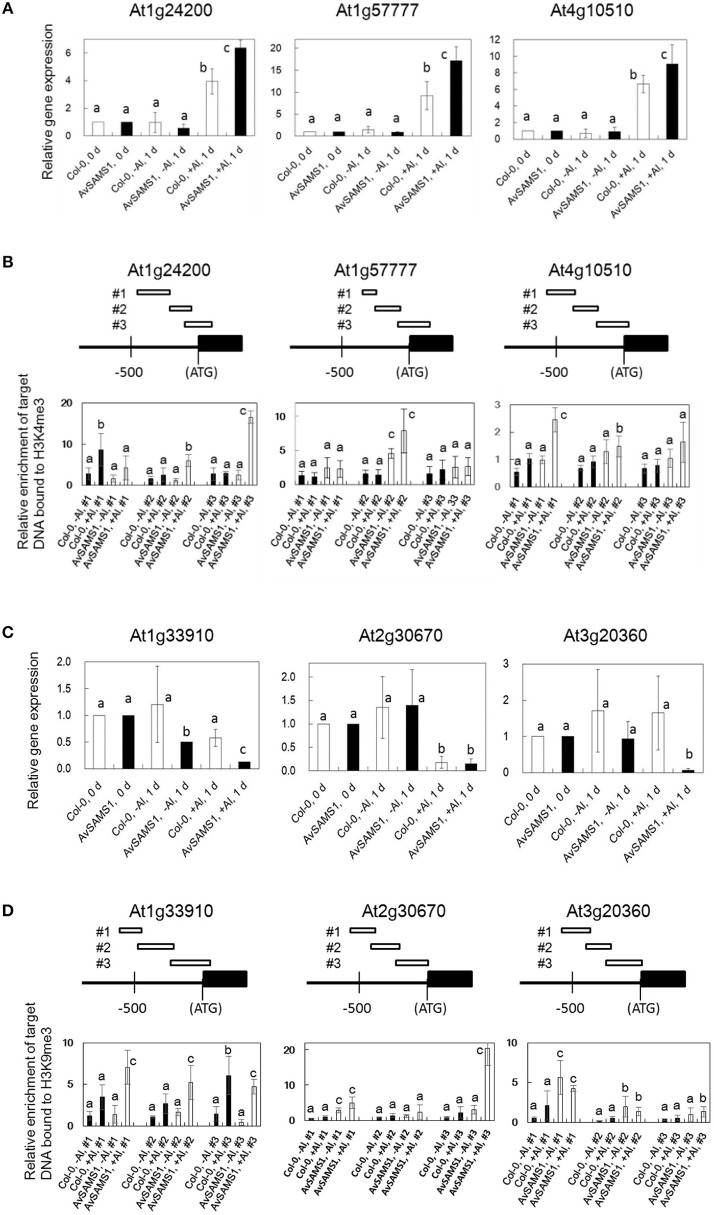
**Alteration of the enrichment of the DNA fragments bound to methylated Histone H3 (H3K4me3 or H3K9me3) under Al stress. (A,B)** Results of Al-induced genes selected from Gene group I. **(C,D)** Results of Al-repressed genes selected from Gene group R. **(A,C)** Relative expression of each gene under Al stress (0 μM Al for 0 and 1 days, and 300 μM Al for 1 days). Values were normalized to the expression of the *AtAct1* gene and are shown as relative fold expression compared to that seen in Col-0 or *AvSAMS1* TF at 0 days for each condition (set to 1). **(B,D)** Relative enrichment of recovered target-DNAs (three target sites in each tested gene, #1, 2 and 3) in the tri-methylated H3 fractions. DNA/Histone complex including H3K4me3 or H3K9me3 were extracted and prepared from Al treated (300 μM Al treatment for 1 days) or untreated (0 days) plants of Col-0 or from those of the *AvSAMS1* TF. DNA content in each DNA/histone complex was determined by qRT-PCR and then normalized with the *AtAct1* gene content bound to either H3K4me3 or H3K9me3. All qRT-PCR were replicated three times for each sample from three independent ChIP experiments. Error bars indicated SD. Different letters above the bars indicate significant differences among the four values in each DNA fragment (*p* < 0.05).

The relationship between histone 3 (H3) methylation and regulation of gene-expression has been well studied and DNA regions that strongly interact with H3K4me3 or H3K9me3 are generally induced or repressed in their gene-expression levels, respectively (see review, Henderson and Jacobsen, 2007). Is there also a similar relationship in the strength of interaction between genome DNA and methylated histones (H3K4me3 and H3K9me3) under Al treatment? Furthermore, are there any Al-stress-dependent differences in the DNA/methylated histone interaction between Col-0 and the *AvSAMS1* TF line? To address these questions, two types of nucleosome including H3K4me3 or H3K9me3 were individually prepared and precipitated by ChIP method from both Al treated and non-treated plants (Al-treated Col-0, untreated Col-0, Al-treated *AvSAMS*1 TF and untreated *AvSAMS1* TF). Since only DNA fragments specifically bound to these two types of nucleosome are preferentially recovered in this method, alteration of histone methylation status under Al stress can be investigated by the determination of the recovered DNA contents. Fifteen and thirteen genes were selected from “Gene group I” and “Gene group R”, respectively (totally 28 genes, Supplement Table 1) and the contents of the recovered individual DNA fragment was determined by qRT-PCR. Representative results of six genes were shown in Figure 5. Among the tested Al induced genes, At1g24200 (Paired amphipathic helix repeat-containing protein), At1g5777 (Uncharacterized protein) and At4g10510 (Subtilase family protein) were confirmed their higher expressions in the TF than in Col-0 under Al stress (Figure 5A). Compared with -Al condition, one or two of the tested regions of these genes were enriched in the ChIP treated fraction including H3K4me3 under +Al condition (“Al-treated Col-0 vs. untreated Col-0” and “Al-treated *AvSAMS*1 TF vs. untreated *AvSAMS1* TF”), indicating that a part of promoter region was enriched by an interaction with H3K4me3 under Al stress (Figure 5B). Moreover, a higher enrichment of these fragments in the recovered nucleosome fraction was observed in the TF than in Col-0. The members of Al repressed genes, At1G33910 (Putative avirulence-responsive protein), At2G30670 (Tropine dehydrogenase) and AT3G20360 (TRAF-like family protein), were also confirmed their repressed expression patterns by qRT-PCR (Figure 5C). qRT-PCR analysis of these three genes indicated that the either of the three fragments in the promoter region of each gene were enriched by an interaction with the repress type nucleosome including H3K9me3. Moreover, a higher enrichment of DNA fragment was observed in the Al-treated *AvSAMS1* TF line than in the Al-treated Col-0 line (Figure 5D).

## Discussion

To understand the high Al tolerance phenotype of *A. virginicus* L., a direct screening of Al tolerant genes was performed in this study. One of the isolated clones, *AvSAMS1*, was inducible by Al stress and could confer Al tolerance to *A. thaliana*. Since this gene could also confer tolerance phenotypes for Cu, Zn, Pb, and DM, it was suggested to be a multiple tolerance gene for these abiotic stresses. Guo et al. (2014) reported that plant *SAMS* genes are induced by various environmental stresses, such as cold stress, salt stress, Cd stress and so on and suggested that some of them are related to tolerance mechanisms. Rice and tomato *SAMS* genes were also induced by Al stress, but it was not clear whether they were actually related to Al tolerance mechanisms (Yang et al., 2007; Zhou et al., 2009). Recently, Kim et al. (2015) reported that expression of a potato *SAMS* gene (*SbSAMS*) in *A. thaliana* TF plant could confer tolerance to salt stress and drought, and concluded that SAM was produced as a broad-spectrum signal molecule to upregulate stress-related genes. Our results are fully consistent with all of these results, and moreover suggest a relationship of SAM as a methyl-residue donor and SAMS as a regulator affecting the epigenetic regulation of Al stress.

This is the first report that the Al-stress-induced *SAMS* gene derived from *A. virginicus* L. (*AvSAMS1*) can confer tolerance to Al toxicity. It is also interesting that the *AvSAMS1* gene shows a multiple-response in *A. virginicus* L. and a multiple-tolerance to heavy metal stresses and DM-derived oxidative stress in *A. thaliana*. The *AvSAMS1* gene may have a similar tolerance function for all of these stresses. It was originally screened and isolated in yeast as an Al tolerant gene, even though it did not carry approximately 200 bp of its N-terminal region which is well conserved across different species. It has been suggested that this region interacts with its methionine substrate, but the precise binding site was still unclear (Garrido et al., 2011). It is presumed that the partial AL3A-4 subclone when over-expressed in yeast retained at least part of its activity. “Tolerance spectrum” depends on “stress response” in individual organisms, but “response to stress” and “tolerance to stress” is not same each other. For example, the *AvSAMS1* gene is responsible for Al, Cu and DM in *A. virginicus* L. as described in Result (please see Figures 2A,B), but we do not know whether the gene can confer tolerance to individual abiotic stresses in this plant. What is the explanation for the different tolerance to different abiotic stress among yeast, *A. virginicus* L. and *A. thaliana* in this study? A possible explanation for the difference in the tolerance spectrum (especially between *A. thaliana* and yeast in this study) may be that we used different promoters (the *GAL1* prompter for yeast and CaMV 35S promoter for *A. thaliana*) to express the *AvSAMS1* gene in these two organisms. These promoters are furthermore different from the domestic *AvSAMS1* promoter in *A. virginicus* L. The expression-pattern of the *AvSAMS1* gene in *A. virginicus* L. were shown in Figures 2A,B. While, we did not determine the expression level of *AvSAMS1* gene in the *A. thaliana* TFs under various stress conditions, because the gene-expression was expected to be regulated by a constitutively and strongly expressing promoter, CaMV 35S promoter. The expressing level of this gene in yeast TF was also kept high, because galactose was added to the medium and agar plates (as an inducer) to induce the gene-expression during the stress sensitivity tests. It is therefore easily suggested that there are differences in both expression-level and expression-pattern under various stress conditions among yeast, *A. thaliana* and *A. virginicus* L. in our study. These differences in gene-expression of the *AvSAMS1* may effect on the differences in the tolerance spectrum of these three organisms, even though we used the same full length *AvSAMS1* gene. Alternative simple explanation may be that the SAMS dependent epigenetic gene-regulation probably has specificity in its contribution to abiotic stress-tolerance. We think that the epigenetic regulation can effect on some of the abiotic stresses with a degree of difference, but completely not on other stresses. This specificity must be different among organisms and causes a difference in tolerance spectrum in each organism. Tolerance spectrum can be said an important point of view to characterize the biological functions of the *AvSAMS1* gene in the epigenetic gene-regulation under abiotic stress and we would like to address this theme as a future work.

We hypothesized that there was a difference in the SAM contents between the two lines due to an introduction of the *AvSAMS1* gene and that the different SAM contents lead a difference in methylation status of genome DNA and/or histones under Al stress. Genome-wide gene-regulation via the deduced epigenetic regulation therefore might be preferentially enhanced in the *AvSAMS1* TF than Col-0 under Al stress. To confirm our speculation, we performed two experiments. Result of DNA sequencing after bisulphite treatment demonstrated that some genes (e.g., At2g34430 and At4g12230) showed a clear change of DNA methylation status in response to Al stress, and moreover that Col-0 and the *AvSAMS1* TF line individually showed significant differences in their DNA methylation status during Al stress. It was concluded that an epigenetic regulation exists in *A. thaliana* during Al stress and that the *AvSAMS1* gene was able to confer differences in DNA methylation status. It has been well known that DNA methylation causes a down regulation of gene-expression, because a stereo-hindrance inhibits the binding of various transcription factors and RNA polymerase to the methylated DNA region. In this study, some promotion of DNA methylation under Al stress was observed in several Al-induced genes which were the members of in the Gene group I. However, methylation and de-methylation occurred simultaneously and intricately in a narrow area in At2g34430 as well as other tested genes, in both plant lines. In these cases, DNA methylation and demethylation occurred individually in each C site, but not evenly as an area. Recently, it has been reported that DNA methylation occurs in both promoter and gene-body and these methylations individually have different effects on the gene-expression. DNA methylation in promoter regions usually represses gene-expression. However, in the case of the gene-body, a mild methylation promoted gene-expression, but extremely low or high DNA methylations caused a lower gene expression (Suzuki and Bird, 2008; Takuno and Gaut, 2012). In this study, different DNA methylation status in the gene-body was also detected in At4g12230 and caused a difference in Al response between Col-0 and the *AvSAMS1* TF, suggesting a consistence with their reports.

Approximately half of the tested genes in each Gene group showed a non-methylation in all C sites and no change in methylation status by Al treatment in both plants (Table 1, non-ME). Since all of these genes also showed higher or lower expressions by Al stress in the *AvSAMS1* TF than in Col-0, it seemed that these genes did not follow the deduced *AvSAMS1*-dependent epigenetic gene-regulation and that these results were not consistent with our hypothesis. However, it may be that epigenetic control of these genes occurred indirectly, for example via controlled by other transcription factors that are regulated by methylation. An alternative explanation may be that other *AvSAMS1*-dependent methylation systems, such as histone methylation, regulates the expression of these genes under Al stress in the *AvSAMS1* TF line. There may also be additional stress-induced mechanisms controlling gene-expression, so that a combination of the gene-regulation systems decides the final expression level of individual gene under Al stress.

Histone methylation is also very important in epigenetic regulation and has been reported to be related to both gene-induction and gene-repression by the various methylations in the four histone proteins (H2A, H2B, H3, and H4). In this study, we focused on the tri-methylation in H3K4 and H3K9 and investigated the methylation status of these histones under Al stress. We estimated the relative abundance of DNA fragments recovered after ChIP that were specifically bound to nucleosomes carrying either H3K4me3 or H3K9me3, but not to the nucleosome carrying non-methylated H3K4 or H3K9. The alteration of the recovered target DNAs by Al stress indicates the alteration of methylation status of H3K4 and H3K9 by Al stress. This method allows precise investigation of the histone methylation status in individual genes. In this study, the promoter regions of 28 genes selected from Gene group I or R were tested and approximately 30% of them showed both an alteration of enrichment of the DNA contents in the recovered tri-methylated histone complex by Al stress, and a clear difference in the DNA enrichment in the *AvSAMS1* TF line (Figures 5B,D). These results indicated that an alteration of H3 methylation status (in H3K4 or H3K9) occurred by Al stress in *A. thaliana*. Moreover, the two types of H3 methylation preferentially occurred in the *AvSAMS1* TF, suggesting an *AvSAMS1* dependency in the methylation under Al stress. Although approximately 70% of the tested genes showed no changes in tri-methylation of H3K4 or H3K9, epigenetic modifications other than methylation and modifications to the other histone proteins have not been investigated yet. Total methylation status in H3K4, H3K9, or H3K27 under Al stress also should be investigated by western blotting analysis as a future work.

In this study, differentially induced or repressed genes between Col-0 and the *AvSAMS1* TF were selected from Gene group I or R. These two groups were classified from the microarray data and used for further analyses, but the array experiments with the two biological replicates might increase the rate of both false positives and false negatives and might not to be enough to obtain reproducible data for the selection. In this study, we found that the Al dependent alteration of methylation status in DNA and histone are actually occurred in some genes, but not all genes. Moreover, the methylation especially in DNA was very complex and the alterations seemed not to simply follow whether the target gene was Al induced type or repressed type. To understand the relation between the *AvSAMS1* dependent epigenetic regulation and gene-response under Al stress more clearly, integrated analyses in the two lines for alteration of methylation status and for gene expression by microarray must be necessary. Future study will help us to understand how Al stress regulates gene-expressions by epigenetic modification.

## Author contributions

BE, AH, NN, and TN performed the experiments. BE and TN analyzed the data. BE planned the project and wrote the article.

## Funding

This work received financial support from the Ministry of Education, Culture, Sports, Science and Technology (Grant-in-Aid for Scientific Research (C)(2) no. 2358092 and no. 15K07339 to BE), The Yakumo Foundation for Environmental Science (to BE), Wesco Scientific Promotion Foundation (to BE), Ryobi Teien Memory Foundation (to BE) and Ohara Foundation for Agriculture Sciences (to BE).

### Conflict of interest statement

The authors declare that the research was conducted in the absence of any commercial or financial relationships that could be construed as a potential conflict of interest.

## Supplementary material

Supplement Figure 1 **Comparison of the full length of the *AvSAMS1* cDNA (Gene Bank Accession Number, AB907169) and other highly homologous sequences of the four *SAMS* cDNA (KJ577596 derived from *S. hybrid*, BT088116 from *Z. mays* and AK103157 from *O. sativa*)**. The latter three cDNAs were retrieved from DDBJ and then aligned with the *AvSAMS1*. Start codon (ATG) and stop codon (TAA) of each gene were shown with bold red characters. Two symbols “^*^” and “•” represented 100% and 75% identity among the four sequences, respectively.

Supplement Figure 2 **Phylogenetic tree of the *SAMS* genes among plants, yeast and animal**. The tree was created by UPGMA method.

Supplement Figure 3 **Competitive microarray data**. Scatter plot of Col-0 ecotype **(A)** and the *AvSAMS1* TF line **(B)**. Total RNA samples were extracted from seedlings treated either with 300 μM Alor without Al (control; −Al condition) for 2 days at pH 4.2. X and Y axes indicate signal intensities of each gene under −Al and +Al conditions, respectively. Three slopes of pale green lines (right, center and left) in each panel show 4, 1 and 0.25 relative fold changes, respectively.

Supplement Figure 4 **DNA methylation status of At1g17180 (region of 52–314 nt) under A stress in Col-0 and the *AvSAMS1* TF**. Result of the original sequence (ori) and the four bisulphite treated sequences (Col-0, ±Al and the *AvSAMS1* TF, ±Al) were aligned. All C sites in the four sequenced regions were replaced to T indicating that all C were kept as non-methylated style (free form).

Supplement Figure 5 **Alteration of Al stress dependent DNA methylation status caused in the Al response gene, At4g12230**. Raw data of DNA sequence pattern of the four templates extracted from the untreated plants (0 days) (Condition 1 and 3) or from the Al treated plants (300 μM Al treatment for 2 days, +Al)(Condition 2 and 4). Condition 1 and 2, Col-0 ecotype; Condition 3 and 4, the *AvSAMS1* TF line. Symbols shown in the bottom of each sequence pattern represented methylation status at C sites. •, Mixture of methylated C and non-methylated C; •, non-methylated C only; •, methylated C only. The limit for defining mixtures was taken as a ratio of >0.1 up to <0.9. Two DNA sequences in the middle were the original sequence and the bisulphite treated DNA sequence of this gene. Y with red color in the latter sequence represents a mixture of C and T in the sequence, indicating both methylated C (retained as C after bisulphite treatment) and non-methylated C (converted to T by bisulphite treatment).

Supplement Table 1 **List of genes used for analysis of histone methylation status**.

## Abbreviations

ChIP: Chromatin immuno-precipitation
DM: diamide
H3K4me3: tri-methylated H3 in the 4th Lys residue
H3K9me3: tri-methylated H3 in the 9th Lys residue
qRT-PCR: quantitative real time PCR
RACE: rapid amplification of cDNA ends
SAM: S-adenosyl methionine
SAMS: S-adenosyl methionine synthetase
TF: transformant.
