# Supplementary figures and images for: An S-adenosyl Methionine Synthetase (SAMS) Gene from Andropogon virginicus L. Confers Aluminum Stress Tolerance and Facilitates Epigenetic Gene Regulation in Arabidopsis thaliana
Source: Front Plant Sci. 2016 Nov 8;7:1627. doi: 10.3389/fpls.2016.01627 (PMC5099669; doi:10.3389/fpls.2016.01627)

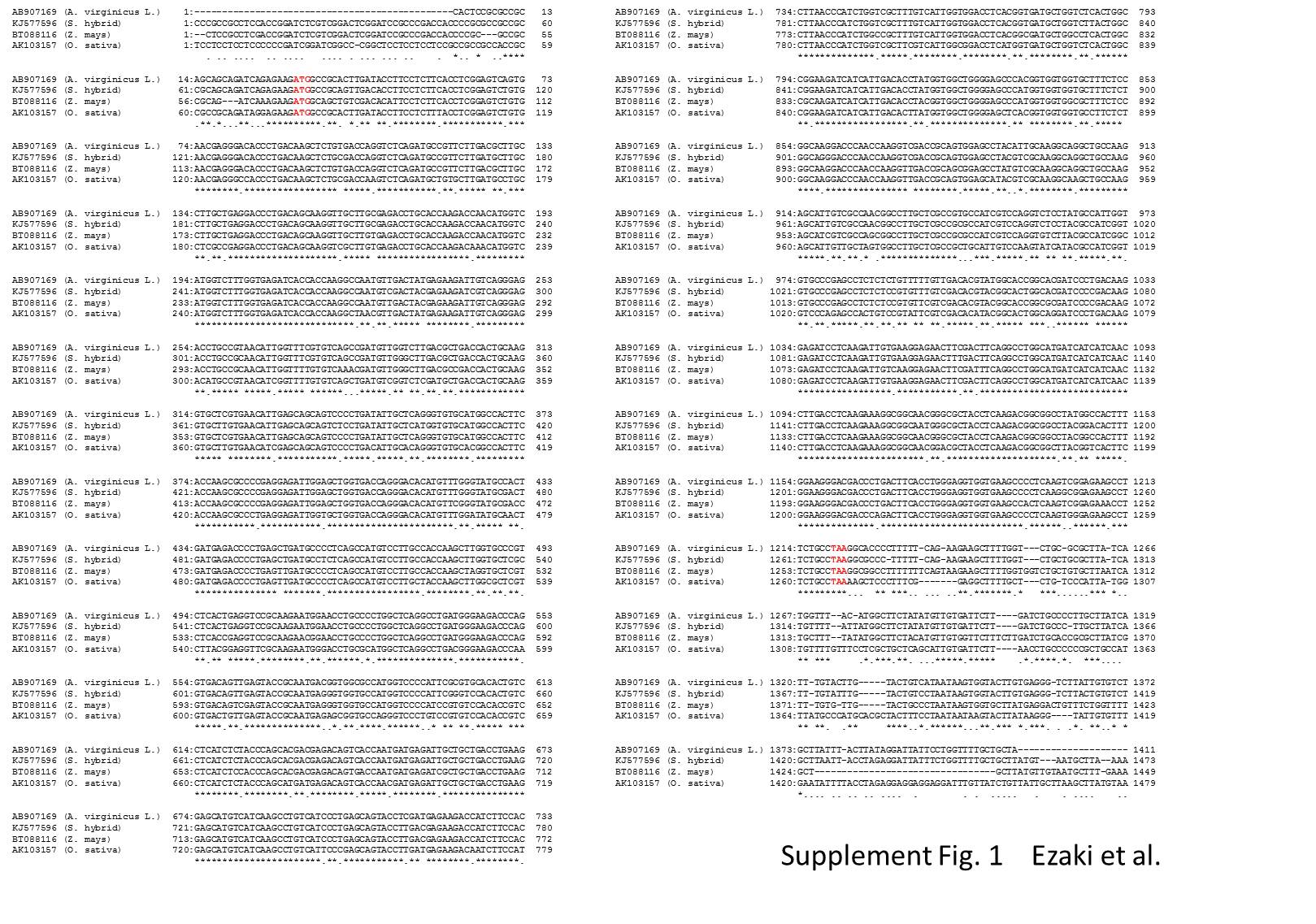

Supplement: Supplement Figure 1 — Comparison of the full length of the AvSAMS1 cDNA (Gene Bank Accession Number, AB907169) and other highly homologous sequences of the four SAMS cDNA (KJ577596 derived from S. hybrid, BT088116 from Z. mays and AK103157 from O. sativa). The latter three cDNAs were retrieved from DDBJ and then aligned with the AvSAMS1. Start codon (ATG) and stop codon (TAA) of each gene were shown with bold red characters. Two symbols “*” and “•” represented 100% and 75% identity among the four sequences, respectively. [file Image1.JPEG]

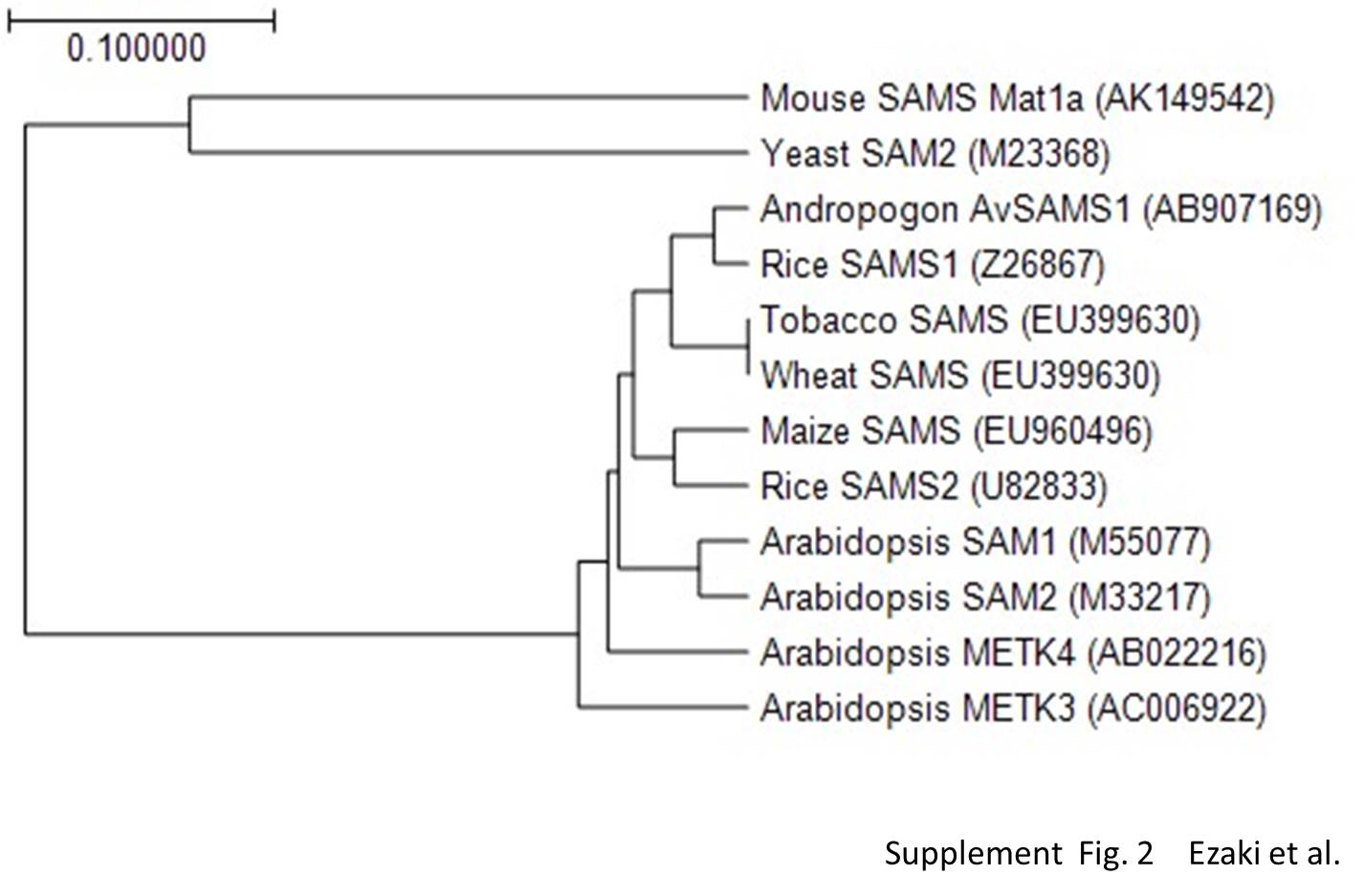

Supplement: Supplement Figure 2 — Phylogenetic tree of the SAMS genes among plants, yeast and animal. The tree was created by UPGMA method. [file Image2.JPEG]

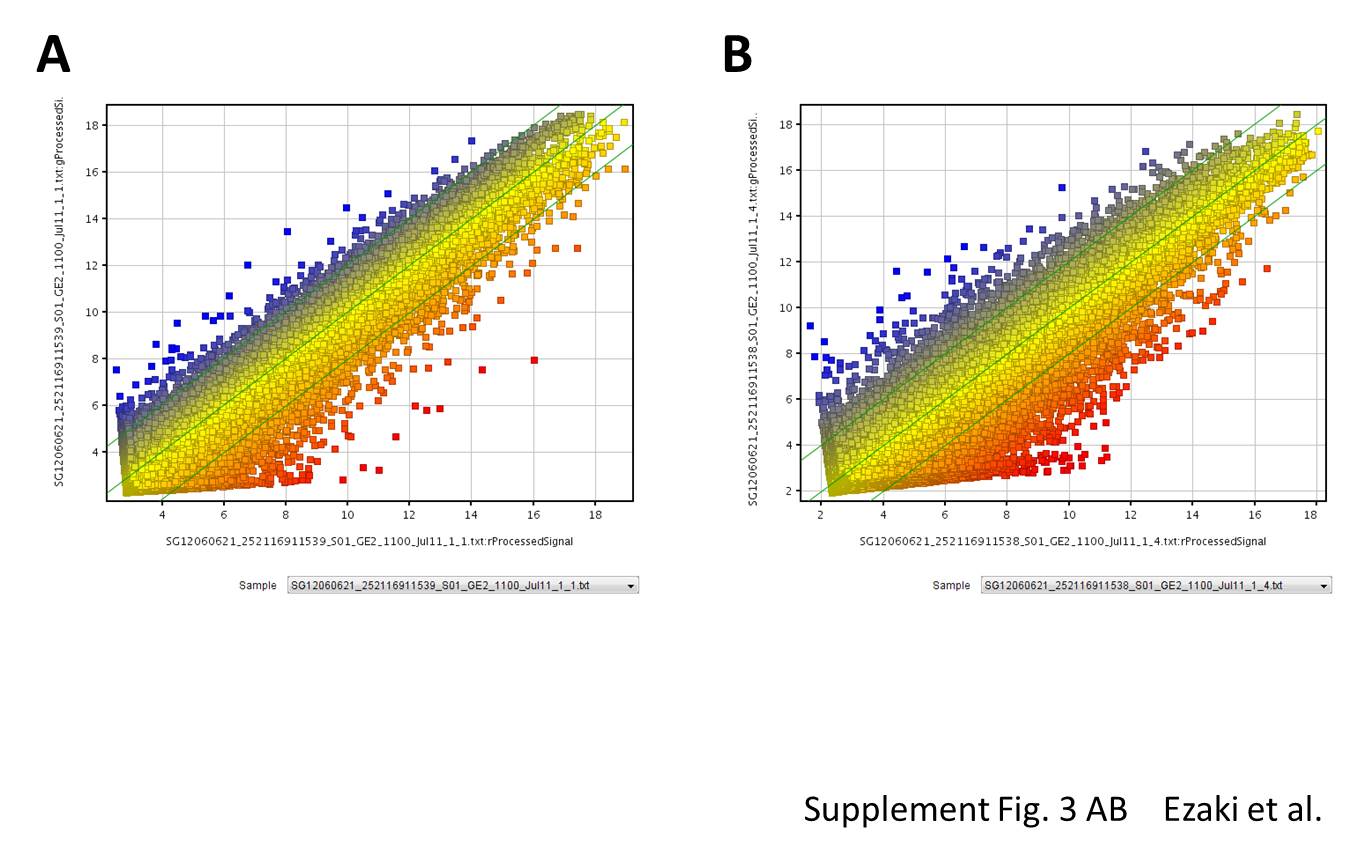

Supplement: Supplement Figure 3 — Competitive microarray data. Scatter plot of Col-0 ecotype (A) and the AvSAMS1 TF line (B). Total RNA samples were extracted from seedlings treated either with 300 μM Alor without Al (control; −Al condition) for 2 days at pH 4.2. X and Y axes indicate signal intensities of each gene under −Al and +Al conditions, respectively. Three slopes of pale green lines (right, center and left) in each panel show 4, 1 and 0.25 relative fold changes, respectively. [file Image3.JPEG]

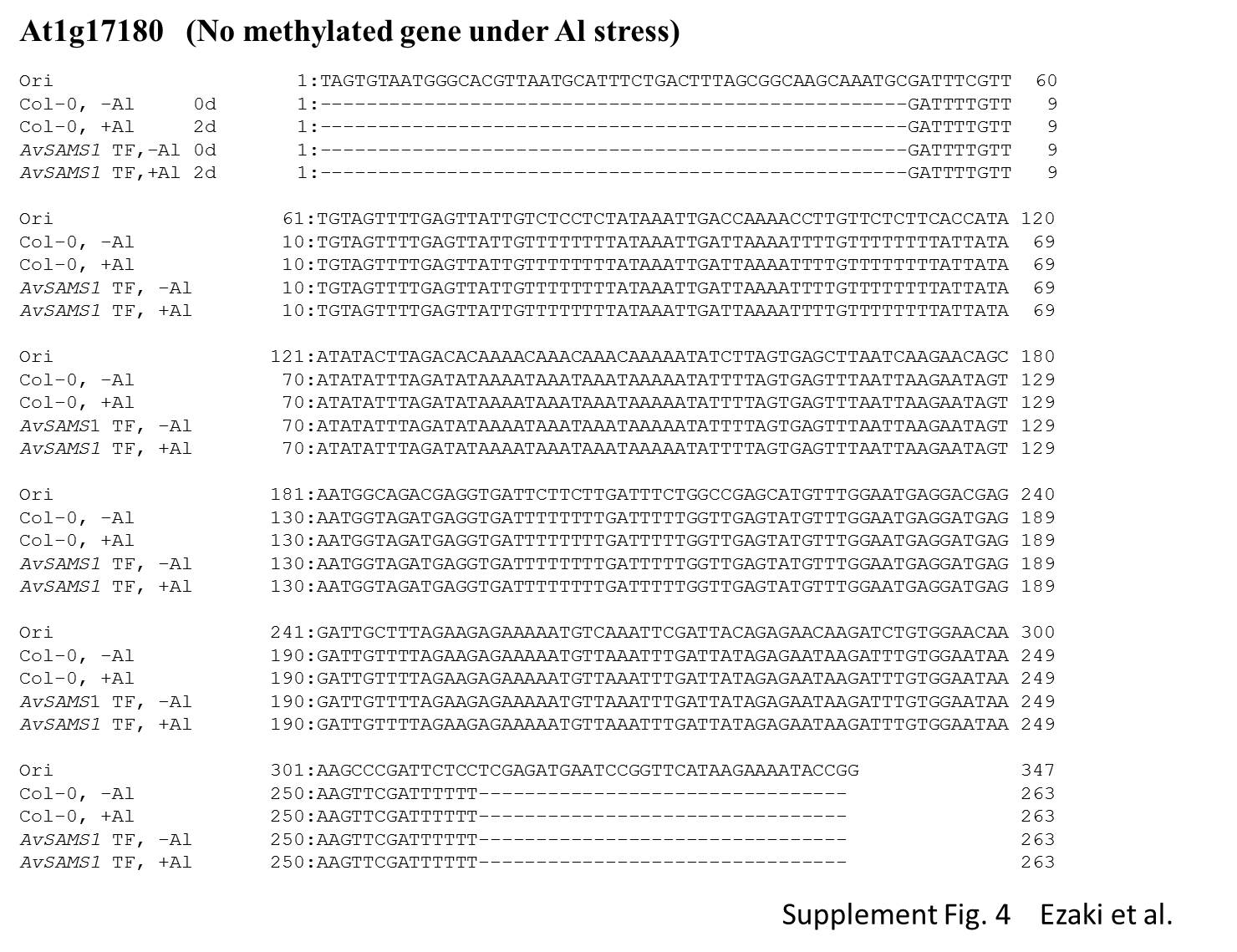

Supplement: Supplement Figure 4 — DNA methylation status of At1g17180 (region of 52–314 nt) under A stress in Col-0 and the AvSAMS1 TF. Result of the original sequence (ori) and the four bisulphite treated sequences (Col-0, ±Al and the AvSAMS1 TF, ±Al) were aligned. All C sites in the four sequenced regions were replaced to T indicating that all C were kept as non-methylated style (free form). [file Image4.JPEG]

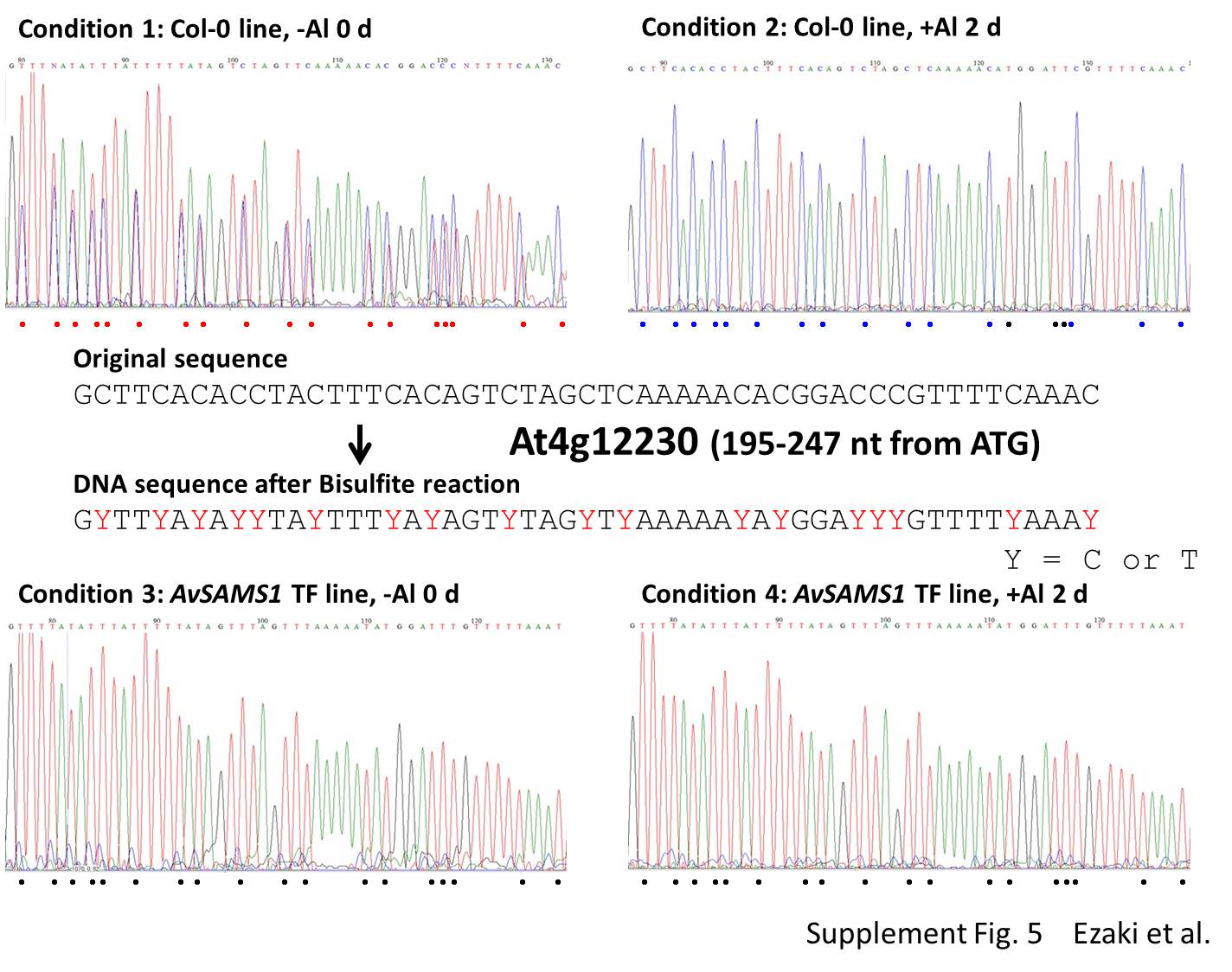

Supplement: Supplement Figure 5 — Alteration of Al stress dependent DNA methylation status caused in the Al response gene, At4g12230. Raw data of DNA sequence pattern of the four templates extracted from the untreated plants (0 days) (Condition 1 and 3) or from the Al treated plants (300 μM Al treatment for 2 days, +Al)(Condition 2 and 4). Condition 1 and 2, Col-0 ecotype; Condition 3 and 4, the AvSAMS1 TF line. Symbols shown in the bottom of each sequence pattern represented methylation status at C sites. •, Mixture of methylated C and non-methylated C; •, non-methylated C only; •, methylated C only. The limit for defining mixtures was taken as a ratio of >0.1 up to <0.9. Two DNA sequences in the middle were the original sequence and the bisulphite treated DNA sequence of this gene. Y with red color in the latter sequence represents a mixture of C and T in the sequence, indicating both methylated C (retained as C after bisulphite treatment) and non-methylated C (converted to T by bisulphite treatment). [file Image5.JPEG]

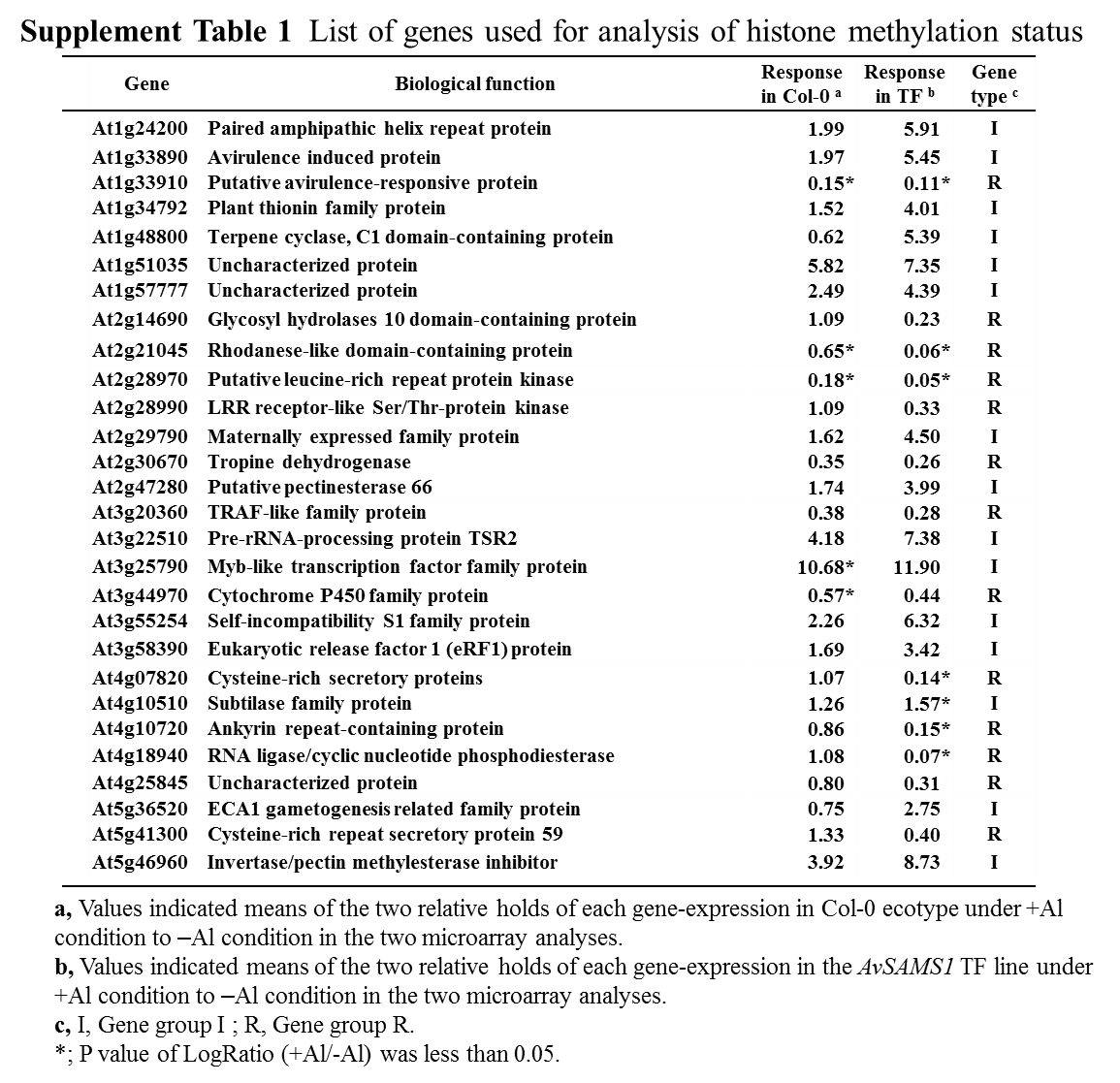

Supplement: Supplement Table 1 — List of genes used for analysis of histone methylation status. [file Table1.docx]
